# Response to “Low cost, uncertain value: Why cheap PV may still not become UK’s main power source”

**DOI:** 10.1016/j.patter.2023.100753

**Published:** 2023-05-12

**Authors:** Filip Mandys, Mona Chitnis, S. Ravi P. Silva

**Affiliations:** 1 School of Economics, University of Surrey, Guildford, UK; 2 Research & Market Analysis Division, European Investment Fund, Luxembourg, Luxembourg; 3 Research Institute for Labour and Social Affairs, Prague, Czech Republic; 4 Surrey Energy Economics Centre, School of Economics, University of Surrey, Guildford, UK; 5 Advanced Technology Institute, University of Surrey, Guildford, UK

## Abstract

We endorse the ideas presented by the authors Klöckl et al. on the need of a mixed bag of energy supplies, with this likely being a combination of solar, wind, hydro, and nuclear in the future. Nevertheless, based on our analysis, we believe that the increased deployment capacity of solar photovoltaic (PV) systems will decrease their cost more than wind, making solar PV important in meeting the Intergovernmental Panel on Climate Change (IPCC) requirements for greater sustainability.

## Main text

We would like to thank Klöckl et al.^1^ for their comments on our recent paper “Levelized cost estimates of solar photovoltaic electricity in the United Kingdom until 2035.”^2^ We fully endorse their ideas and, much like the authors, agree with the need of a mixed bag of energy supplies. Our analysis is based on evidence, and in our view the increased capacity of deployment of solar photovoltaic (PV) systems will further decrease its cost. Wind energy has already seen the benefits of increased deployment and thus will also see a cost drop but to a lesser extent.^3^^,^^4^ Solar PV systems also benefit from the advantage of having the ability to be planned and established swiftly in comparison to other large-scale utility energy sources. There is little time to be lost if we are to reach net zero by 2050.^5^

In all scenarios, we are of the opinion that renewable energy systems will need to have large-scale energy storage, with a minimum baseline energy provision that will prevent blackouts and brownouts. In the future, this is likely to be a mix of solar, wind, hydro, and nuclear, linked to distributed energy storage on a large scale within micro-grids. Within the UK, wind energy in the north and solar energy in the south will provide the best mix of green renewables that has the potential to overcome some of the seasonal and daily supply-demand peak offsets needed for a truly versatile and inexpensive micro-grid, reinforced with hydro or other energy storage provisions.^6^

The levelized cost of electricity (LCOE) we have examined does take the total “levelized” cost of electricity provision to account, including load-factors, intermittency, penetration of energy sources, long-term meteorology, and latitude-based variations within the UK. Ultimately it is the data obtained based on evidence of energy cost that will determine the accuracy of the predictions, and we all agree that renewable sources are key to meeting the Intergovernmental Panel on Climate Change (IPCC) requirements for the world to become more sustainable (e.g., Bogdanov et al.,^7^ Holechek et al.,^8^ and Jafari et al.^9^). So, whether it is solar or wind that provides that backbone is a moot point, and what matters is ensuring a mixed renewable energy provision to provide a sustainable and secure energy future.^6^^,^^10^
